# Job Maintenance by Supported Employment: An Overview of the “Supported Employment Plus” Trial

**DOI:** 10.3389/fpubh.2015.00140

**Published:** 2015-05-26

**Authors:** Wolfram Kawohl, Jörn Moock, Sandra Heuchert, Wulf Rössler

**Affiliations:** ^1^Competence Tandem Integrated Services, Innovation Incubator, Leuphana University Lüneburg, Lüneburg, Germany; ^2^Department of Psychiatry, Psychotherapy, and Psychosomatics, Center for Social Psychiatry, Psychiatric Hospital, University of Zurich, Zurich, Switzerland; ^3^University of Zurich, Zurich, Switzerland

**Keywords:** supported employment, job maintenance, individual placement and support, mental health, sickness absence

## Abstract

The number of days of absence from work associated with mental illness has risen dramatically in the past 10 years in Germany. Companies are challenged by this issue and seek help for the physical and mental health of their employees. Supported Employment concepts such as the Individual Placement and Support (IPS) model have been designed to bring jobless persons with mental disorders back to work. In the randomized, controlled SEplus trial, a modified IPS-approach is tested concerning its ability to shorten times of sick leave of persons with mental distress or a mental disorder and to prevent them from losing their job. The trial is outlined in this study protocol.

## Introduction

The number of days of absence from work caused by mental illness has risen dramatically in Germany over the past 10 years. From 2002 to 2012, this number has increased more than 60% (1), with a current average amount of almost 36 absence days per case. In 2012, absence caused by mental illness entailed costs amounting to € 6 billion (2). Although the group of “musculoskeletal and connecting tissue illnesses” is the most often stated cause of sickness absence [23.4%; (2)], theses illnesses are frequently accompanied comorbid mental disorders. Such comorbidities are often neglected by general practitioners and company physicians (3).

To date, only little research has investigated the influence of labor conditions on mental health of members of staff and how to improve employees’ psychological well-being. Buzz words like “burn out” or “total exhaustion” often oversimplify the complex interrelations between the job and an employee’s psyche. Even most social services departments of multinational corporations have only limited experience with mental illnesses in the context of work-related problems and stress. Sick leave due to mental illness is also the most common cause for claiming government pension due to a reduction of earning capacity (4). However, there seems to be an imbalance between the importance of applied prevention tools and the amount of research conducted to systematically evaluate these tools.

With the present study, we aim to reduce this gap in the current body of literature and assess the effectiveness of a supported employment concept, which seeks to reduce work-related mental strain and aims to re-establish and maintain the mental health of employees. Past research indicates that supported employment concepts, such as the Individual Placement and Support approach [IPS; e.g., Ref. (5)], contribute to the successful reintegration of unemployed persons with mental disorders (6). However, as IPS has been conceptualized to bring jobless persons back to work, sparse evidence exists concerning the impact of IPS on job maintenance.

In clinical settings, IPS is already used not only for finding new jobs but also for job maintenance (7). According to a recent meta-analytic study, job coaching has been shown to be effective in organizational contexts (8). Moreover, evidence exists that sickness absence can be reduced by coachings both for employees having been identified as being at risk for sickness absence and their supervisors (9). The main aim of our project is to control the ability of IPS to maintain the jobs of persons who feel psychologically strained or who already have developed a mental disorder and to shorten the amount of absence days. In contrast to the trial of Duijts, the intervention is person-centered and does not automatically include the supervisor of the target persons.

The name “SEplus” has been chosen as the study test an intervention representing an extension of the well documented and researched approach “IPS” (5) as a form of supported employment. The intervention targets employees who are hampered in the proper execution of work-related tasks due to mental problems strain mental disorders. Adverse effects of strain can appear, for example, in the form of decreased performance, increased error rates, a lack of concentration, conflicts with colleagues or superiors, frequent short periods of sickness absence or even long-term absence that may ultimately lead to losing one’s job. The intervention focuses on the re-establishment of employees’ working capacities under consideration of existent resources.

This job coaching is conducted by a special trained job coach and provides a safe and professional setting for the employees to work on the causes of their mental strain or illness. The job coach facilitates and mirrors different perspectives of the employee’s situation and thereby helps to analyze the problem and find a proper solution.

## Hypotheses

Based on the currently available literature, we derived and will test the following main hypotheses:

Job coaching based on the approach of IPS contributes to job maintenance of employees who feel mentally strained or have a manifest mental disorder. Consequently, we hypothesize that this kind of job coaching reduces the number of days of sick leave.

In addition, we hypothesize the following:

Job coaching improves (a) job satisfaction, (b) global functioning, (c) quality of life, and (d) participants’ recovery orientation. Moreover, we assume job coaching to reduce (a) perceptions of self-stigma and (b) cognitive appraisal of stigma as a stressor.

## Materials and Methods

### Study design

The present study is a randomized controlled trial and uses a one-factorial design with two levels (job coaching intervention vs. control group). After having given their informed consent, participants were randomly assigned to the intervention group or to the control group by a Bernoulli randomization. The intervention group received the employee centered job coaching. Participants of the control group were given a self-study brochure explaining how to cope best with mental strain in the workplace. No stratification with respect to company was carried out.

### The job coaching intervention

The job coaching model is adapted from the IPS; (5). However, while IPS supports the reintegration of recovered psychiatric patients into the job market, the job coaching in our trial is applied considerably earlier. The coaching is individually fitted to each employee’s needs and requirements and tackles their specific work-related problems. In the course of the intervention, the job coach provides assistance to the employees to help themselves and regularly evaluates the goals agreed on. Each employee usually attends between 10 and 12 job coaching sessions over a period of 3–4 months.

### Time scale

The intervention has been carried out from November 2012 until September 2014. The evaluation that the data set is currently in progress. Prior to their random allocation to the control or the intervention group, all participants were interviewed and completed a comprehensive set of questionnaires (time point T0; see Table 1). After 3 months (T1), participants of both groups completed a condensed set of questionnaires. T1 usually represents the end of the individual job coaching for participants of the intervention group. Three months following T1, T2 data are collected, using the same questionnaire-based interview conducted at T0.

**Table 1:** **Overview of questionnaire based instruments and when they were administered**.

Instrument	Variable/construct	Perspective	Time of measurement		
			T0	T1/T1a	T2
Demographic questionnaire (Team Supported Employment PLUS, 2012)	Demographic data	P/I	×	–	–

Job Preferences (ZHEPP; University Hospital for Psychiatry Zurich, ZHEPP, 2011)	Job preferences	P/I	×	–	–

Arbeitsbezogenes Verhaltens – und Erlebensmuster [work related behavioral and sensational patterns] [AVEM 44 (10)]	work related experiences and behavior	P	×	×	×

Indiana Job Satisfaction Scale [IJSS (11)]	Job satisfaction	P	×	×	×

Anreizfokus Skala [Incentive focus scale] (12, 13)	Motivation of participant	P	×	×	×

Maslach Burnout Inventory General Survey [MBI GS (14, 15)]	Emotional exhaustion, Depersonalization, Personal accomplishment	P/I	×	×	×

Manchester Short Assessment of Quality of Life [MANSA (16)]	satisfaction with life as a whole and with life domains	P	×	×	×

SCL 90 R [Symptom Checklist 90 Revised (17, 18)]	Psychological symptoms	P	×	×	×

General Health Questionnaire [GHQ, 12 Items (19, 20)]	psychological well-being	P	×	–	×

Scham [Shame] (21)	Experienced shame due to being psychologically strained	P/I	×	–	×

Stress coping model of mental illness stigma [SSMIS (22, 23)]	Emotional stress reactions and cognitive coping responses to stigma	P	×	–	×

Stigma stress (24)	Emotional strain due to stigma	P	×	–	×

Recovery Assessment Scale [RAS, Giffort et al. (25)]	How they feel about themselves and their life	P	×	–	×

Center for Epidemiologic Studies Depression Scale [CES D (26, 27)]	Depressive symptoms	P	×	–	×

LUNST (28)	Social integration	P/I	×	–	×

Work Design Questionnaire [WDQ (29, 30)]	Workplace analysis	P/I	×	–	×

Mini International Classification of Functioning, Disability and Health [Mini ICF (31)]	Description of (qualified) functioning	I	×	×	×

Global Assessment of Functioning Scale [GAF (32, 33)]	Description of (qualified) functioning	I	×	×	×

*P, participant; P/I, participant by communicating with interviewer; I, rating by interviewer*.

Table 1 shows which measurements were administered at the different points in time as well as the perspectives from which the measurements were completed (participant, participant by communicating with interviewer, rating by interviewer).

### Inclusion criteria

Employees are eligible to participate in the study by fulfilling the following criteria:

- voluntariness;
- being willing and able to give informed consent;
- self-reported psychological stress or mental disorder;
- employment at a cooperation partner (e.g., business enterprise or public service) of the SEplus trial;
- working age (18–67 years).

### Exclusion criteria

Employees had to be excluded from our study when they fulfilled one of the following criteria:

- persons who need a psychiatric inpatient treatment at the time of the study;
- persons with an imminent risk of suicide.

### Recruitment

In order to recruit participants, 280 corporations, public and private organizations were contacted. These firms represented potential cooperation partners and were selected based on their geographical location (northern Germany) and their number of employees (over 250). The responsible human resource or health managers were sent an information brochure in order to inform them about the SEplus trial and were asked whether they are interested to participate. In some cases, additional personal meetings took place in order to provide the interested firms with more detailed information about the project before they reached a final decision.

Thirteen firms decided to join the project and enabled their members of staff to participate. Within each firm the project has been announced to employees by the company physicians, employee committees, and the general management. Additionally, the project has been advertised via information emails sent to employees and laid out flyers, which contained all necessary contact details. Interested employees could then call or email the project manager or one of the four job coaches for further information or to directly sign up for participation. Before an appointment was made for the first interview, participants were randomly assigned either to the control or the intervention group.

### Sample size calculation

A calculation of the targeted sample size has been conducted with the following parameters: it is aimed to reduce the number of days of sickness absence due to mental strain or mental disorder through the provision of job coaching by 30%. We assume a statistical power coefficient of 0.8, an alpha level of 0.05, and an effect size of 0.3, which denotes according to Cohen (34), a small to moderate effect size. Moreover, a dropout rate of 20% across all time points of measurement is taken into account. With these parameters, a sample size of 108 participants is required in order to detect a statistical significant effect of the job coaching.

### Sample

In total, 99 participants completed the initial interview at T0. Of these participants, 62.6% were female, 73.7% worked full time, 90.9% worked in a team, and 22.2% held an executive position. Participants had a mean age of *M * = 44.56 years (SD* * = 9.25 years) and were on average 16.82 days absent (SD = 23.77) in the past 6 months.

Seventy-six participants completed the interview with the condensed set of questionnaires at T1 (59.2% female, mean age *M* = 45.38 years, SD = 9.21 years). Compared to T0, 14 participants have entirely dropped out and it has not been possible to schedule an appointment after 3 months with 9 participants. These nine participants, however, remained available for the interview at T2. Thus, 85 participants completed the follow up interview at T2 (61.20% female, mean age *M* = 45.18 years, SD = 9.10 years). Following the intention to treat approach [see Ref. (35)], we intend to carry the T0 data of the participants forward, who had not completed the T1 or the T2 interview.

### Questionnaire measurements

The study included the following questionnaire measures to assess participants’ psychological well-being, satisfaction and work-related aspects:

- job preferences (ZHEPP; University Hospital for Psychiatry Zurich, ZHEPP, 2011);
- Arbeitsbezogenes Verhaltens- und Erlebensmuster [work related behavioral and sensational patterns] [AVEM 44 (10)];
- Indiana Job Satisfaction Scale [IJSS (11)];
- Anreizfokus Skala [Incentive focus scale] (12, 13);
- Maslach Burnout Inventory General Survey [MBI GS (14); German version (15)];
- Manchester Short Assessment of Quality of Life [MANSA (16)];
- SCL-90-R [Symptom Checklist-90-Revised (17); German version (18)];
- General Health Questionnaire [GHQ, 12 Items (19); German version (20)];
- Scham (Shame) (21);
- Center for Epidemiologic Studies Depression Scale [CES D (26); German version (27)];
- Social network and support: LUNST-scales (28);
- Work Design Questionnaire [WDQ (29); German version (30)];
- Mini International Classification of Functioning, Disability and Health [Mini ICF (31)];
- Global Assessment of Functioning Scale [GAF (32); German version (33)];

In addition, data have been also collected regarding stigma and recovery from mental illness. To this end, we included the following measurements in the interviews:

- stress coping model of mental illness stigma [SSMIS (22); short version (23)];
- Stigma stress (24);
- Recovery Assessment Scale [RAS (25)];

### Analysis strategy

First, we will assess whether job coaching has had any effect on the different measured outcome criteria within the intervention group compared to the baseline level prior to the start of the intervention. More specifically, these outcome criteria include days of sickness absence as main outcome, (job) satisfaction, global functioning and perceptions of stigma. We will then compare the development of these criteria over time in the intervention group with the development in the control group. This is done to evaluate whether job coaching yields incremental benefits over the no intervention alternative.

### Ethics

The present research study has been approved by the ethics committee of the Leuphana University of Lunenburg and is carried out in accordance with the declaration of Helsinki. It is officially registered under the International Standard Randomized Controlled Trial Number ISRCTN02422335^1^.

## Discussion

Statistical data of dropping temporarily out from work due to mental strain and psychiatric illness indicate an alarming increase over the past years in Germany (36). Concerning vocational rehabilitation, there is considerable evidence showing that IPS programs significantly contribute to successful reintegration in the open job market (6, 37). However, there is only little research so far, which evaluates the effectiveness of IPS-based interventions that address job maintenance instead of reintegration. With the present study, we aim to make a contribution to fill this research gap. If the results of our analyses support our hypotheses, IPS-based job coaching may qualify as valuable tool even for early stages of mental distress or mental disorders at the work place. This would not only benefit the affected employees but also ultimately result in enhanced productivity and lowered costs related to sick leave of employees.

There are some limitations concerning the design of the study. First, the sample size is smaller than the sample size initially calculated. This may lead to a larger standard error and an underestimation of effects reported. A larger sample size would also allow better adjustments for confounders such as gender, age, work environment, etc. A recruitment of more participants was not possible due to the end of the funding of the study. Second, it cannot be quantitatively controlled for the activities of the control group concerning keeping a job. The mere handing out of a brochure does not balance the coaching of the intervention group.

## Author Contributions

WK, JM, and WR designed the study. SH coordinated the intervention and the data collection. All drafted the manuscript.

## Conflict of Interest Statement

The authors declare that the research was conducted in the absence of any commercial or financial relationships that could be construed as a potential conflict of interest. The Associate Editor Hans Joachim Salize declares that, despite having published with Wulf Rössler, the review process was handled objectively and no conflict of interest exists.

## Footnotes

^1^http://www.isrctn.com/ISRCTN02422335
